# Early Postnatal Administration of Erythropoietin and Its Association with Neurodevelopmental Outcomes and Incidence of Intraventricular Hemorrhage and Hypoxic-Ischemic Encephalopathy: A Four-Week Observational Study

**DOI:** 10.3390/pediatric16020030

**Published:** 2024-04-28

**Authors:** Oana Cristina Costescu, Aniko Maria Manea, Eugen Radu Boia, Daniela Mariana Cioboata, Florina Marinela Doandes, Ileana Enatescu, Sergiu Costescu, Mihaela Prodan, Marioara Boia

**Affiliations:** 1 Department of Neonatology, “Victor Babes” University of Medicine and Pharmacy Timisoara, Eftimie Murgu Square 2, 300041 Timisoara, Romania; costescu.oana@umft.ro (O.C.C.); cioboata.daniela@umft.ro (D.M.C.); doandes.florina@umft.ro (F.M.D.); enatescu.ileana@umft.ro (I.E.); boia.marioara@umft.ro (M.B.); 2 Doctoral School, “Victor Babes” University of Medicine and Pharmacy Timisoara, Eftimie Murgu Square 2, No. 2, 300041 Timisoara, Romania; mihaela.prodan@umft.ro; 3 ENT Department, “Victor Babes” University of Medicine and Pharmacy Timisoara, Eftimie Murgu Square 2, No. 2, 300041 Timisoara, Romania; eugen.boia@umft.ro; 4 Department of Obstetrics and Gynecology, “Victor Babes” University of Medicine and Pharmacy Timisoara, 300041 Timisoara, Romania; costescu.sergiu@umft.ro; 5 Department of Obstetrics and Gynecology, Oravita City Hospital, 325600 Oravita, Romania

**Keywords:** erythropoietin, neurological development, neonatology, newborns

## Abstract

This study aimed to investigate the impact of early erythropoietin (EPO) administration on the neurodevelopment of newborns, specifically focusing on its effects on hypoxic-ischemic encephalopathy (HIE) and intraventricular hemorrhage (IVH). The primary objective was to determine whether early EPO administration could impact the short-term neurodevelopmental outcomes and provide safety in neonates at risk for neurodevelopmental disorders. Conducted at the “Louis Turcanu” Children’s Emergency Clinical Hospital in Timisoara, Romania, this observational study included 121 neonates receiving EPO and 130 No EPO controls. EPO was administered within the first 48 h of life, with doses of 1000 U/kg that escalated to 2000 U/kg if necessary. Besides observing the occurrence of IVH and HIE, this study measured clinical and biochemical markers, including LDH, blood glucose, urea, creatinine, CPK, CRP, PCT, and erythropoietin levels alongside hematology and coagulation profiles. There were no significant differences in baseline characteristics between the groups. The EPO group showed significant reductions in LDH levels from days 1–3 to 7–10 (695.0 U/L to 442.0 U/L) and the APTT value (54.0 s) compared with the No EPO group (38.0 s). Notably, early EPO administration was associated with a significant decrease in HIE severity (beta coefficient: −0.38, *p* = 0.001). Additionally, lower gestational ages and hemoglobin levels correlated with increased severity of HIE. By week four, there was a significant reduction in moderate and severe HIE cases in the EPO group compared with controls (*p* = 0.001). Early administration of EPO in neonates significantly reduced the severity of IVH and HIE, suggesting its potential as a neuroprotective agent in neonatal care.

## 1. Introduction

Erythropoietin (EPO), a glycoprotein hormone primarily known for its role in erythropoiesis, has recently garnered attention in the neonatal medical community for its potential neuroprotective effects [1,2]. EPO is produced in response to hypoxia and regulates red blood cell production. However, studies have indicated that EPO also plays a critical role in the brain’s response to injury, particularly in the context of neonatal care, potentially influencing the neurological development early and later in life by providing a neuroprotective role [3,4].

The neurological development of newborns is a complex process that is susceptible to a variety of insults, including hypoxia-ischemia, inflammation, and hemorrhage [5,6,7]. Complications such as intraventricular hemorrhage (IVH) are more prevalent in preterm infants due to the fragility of their cerebral blood vessels [8] which, even though not being easily preventable due to very early onset peri- and postpartum, are hypothesized to cause less harm in the context of early EPO administration [9,10]. Globally, it is estimated that among neonates who survive the early neonatal period, 52% of those born before 28 weeks, 24% born between 28 and 31 weeks, and 5% born between 32 and 36 weeks experience varying levels of neurodevelopmental impairment [11], while a proportion of approximately 5–10% complicate with IVH or hypoxic-ischemic encephalopathy (HIE) [12]. Therefore, the neuroprotective factors are important to identify from both wellbeing and economical perspectives.

This high incidence of complications highlights the need for effective neuroprotective strategies in neonatal care. EPO, with its multifaceted roles in neuroprotection, angiogenesis, and anti-inflammation, has emerged as a promising candidate [13,14]. A landmark study published in 2016 demonstrated that high-dose EPO administration in preterm infants resulted in improved neurodevelopmental outcomes at 24 months [15]. These findings have been corroborated by animal studies, which have shown that EPO can mitigate brain injury in neonatal models of hypoxia-ischemia [16].

Despite these promising results, the use of EPO in neonatal care is not without challenges. The optimal dosing, timing, and duration of EPO therapy remain under investigation. Additionally, concerns regarding the potential long-term effects of EPO, such as the risk of retinopathy in preterm infants, necessitate a cautious approach [17,18,19]. Thus, a meta-analysis indicated that while short-term outcomes are promising, long-term safety data are still required [20]. The role of EPO in modulating other aspects of neonatal brain development, such as synaptic plasticity, neurogenesis, and myelination, is also an area of active research [21,22,23].

Given the existing evidence and ongoing research, this study aimed to explore the associations between early erythropoietin administration (within 48 h post-birth) and the progression of HIE and IVH in newborns during the first four weeks post-treatment. Moreover, we aimed to evaluate the impact of EPO on short-term neurodevelopmental outcomes and biochemical markers in neonates at risk for neurological impairments, as well as assess the safety and physiological responses to early EPO administration in the context of neonatal care, focusing on clinical parameters such as APGAR scores, hematological profiles, and coagulation factors, as secondary study outcomes.

## 2. Materials and Methods

### 2.1. Design and Ethics

This retrospective observational study included a cohort of preterm and full-term neonates admitted to a specialized neonatal care unit at the “Louis Turcanu” Children’s Emergency Clinical Hospital in Timisoara, Romania; the aim was to explore any potential correlations between early EPO administration and the biochemical markers as well as to determine the effect of early EPO administration on the progression of HIE and IVH. Data retrieval was performed from November 2021 to December 2023 from electronic and paper records of the patients. This study was conducted in accordance with the ethical standards of the institutional research committee and with the 1964 Helsinki declaration and its later amendments of ethical standards. The study protocol was reviewed by the ethics committee of the affiliated hospital and approved on 29 November 2023 with approval number 121. Informed parental consent was obtained from all individual parents of the participants included in the study.

### 2.2. Study Population and Inclusion Criteria

The study focused on a specific cohort of neonates, encompassing a study group of 121 newborns who received EPO and 130 infants who did not receive EPO, considered as the control group, admitted to the neonatology unit. The inclusion criteria targeted neonates diagnosed with hypoxic-ischemic encephalopathy and intraventricular hemorrhage. The exclusion criteria comprised the following: (1) neonates with cerebral malformations, including infants with structural brain abnormalities that could significantly alter neurological outcomes; (2) neonates with genetic pathologies, thereby excluding infants with known genetic disorders or syndromes that could independently influence neurological development; and (3) neonates with craniovertebral dysraphism, a group of congenital malformations affecting cranial and vertebral bones potentially impacting the central nervous system. These criteria aimed to establish a homogenous study population, essential for accurately assessing the impact of erythropoietin on the neurological development of neonates affected by HIE and IVH.

### 2.3. Study Variables and Protocols

Erythropoietin levels were initially sampled within the first week of life and prior to the initiation of the first dose and before each subsequent dose to ensure the absence of residual exogenous EPO influencing the measurements. The intervention group received early EPO administration within the first 48 h of life, followed by 5 additional doses. The dosage of EPO beta was 1000 U/kg/dose administered subcutaneously with an escalated dose of 2000 U/kg if required. Subsequent doses were given based on the clinical condition of the neonate and the decision of the attending neonatologist under the conditions of low birth weight for gestational age, moderate and severe anemia, or insufficient initial response, which was determined by evaluating hemoglobin levels and clinical assessments. The control group included neonates whose parents either declined or where EPO treatment was not available in the hospital.

This study assessed a comprehensive range of variables for both the study and control groups. These variables included demographic data such as sex, gestational age in weeks, and birth weight. Clinical assessments were conducted, including APGAR scores at 1 and 5 min post-birth. Biochemical markers were extensively measured: lactate dehydrogenase (LDH) levels within the first 1–3 days and 7–10 days; blood glucose levels; urea; creatinine; creatine phosphokinase (CPK); C-reactive protein (CRP) levels at 24 and 72 h; procalcitonin (PCT) levels at 24 and 72 h; and blood pH, PCO2, and PO2 levels. Additional assessments included lactic acid (LAC) levels and erythropoietin (EPO) levels at one and three weeks of life.

This study also monitored and recorded the presence of ventriculomegaly, varying severities of hypoxic-ischemic encephalopathy (HIE), grades of intraventricular hemorrhage, and the administration of EPO. Furthermore, hemoglobin (Hb) levels, hematocrit (HCT) percentage, red blood cell (RBC) count, activated partial thromboplastin time (APTT), prothrombin time (PT), and PT activity were measured. Finally, occurrences of apnea and bradycardia were noted. In the current study, we included neonates whose assessments were performed at two time points, within the first 48 h of life and at 4 weeks of life, to help monitor their physiological status and evaluate their evolution under EPO treatment regarding HIE and IVH.

### 2.4. Reference Values and Definitions

In this study, established reference intervals were utilized for key biochemical markers. LDH levels were considered to be within a normal range if they fell between 135 and 750 U/L [24], and serum EPO levels were deemed normal when they were between 4 and 24 mIU/mL [25]. For categorizing birth weight (BW), this study adhered to WHO guidelines [26], defining neonates weighing less than 1000 g as “extremely low”, those under 1500 g as “very low”, and those below 2500 g as “low”. Gestational age (GA) was also classified following WHO standards [27]. Infants born before 28 weeks of gestation were categorized as “extremely preterm”, those born between 28 and less than 32 weeks as “very preterm”, those between 32 and 34 weeks were considered “moderate preterm”, and those between 35 and 37 weeks were considered “late preterm” newborns. The APGAR scoring system, as defined by the American College of Obstetrics and Gynecology [28], classified scores of 7–10 as “reassuring”, 4–6 as “moderately abnormal”, and 0–3 as “low”. Normal ranges for prothrombin time and activated partial thromboplastin time were considered to be 11–14 s and 23–35 s, respectively [29,30].

In our study, ventriculomegaly was defined using specific cranial ultrasound measurements that included the Levene index, the thalamo-occipital distance (TOD), and the anterior horn width (AHW). These measurements were selected due to their reduced sensitivity to variations in gestational age, providing more consistent indicators across the neonatal cohort. Ventriculomegaly was identified when the AHW exceeded established normative values for the neonatal age group under study. Hydrocephalus was defined as a progressive increase in these ventricular measurements, specifically when sequential ultrasounds showed a marked increase in the size of the ventricles coupled with clinical signs of increased intracranial pressure or neurological deterioration.

### 2.5. Data Analysis

Before the study commencement, the sample size was calculated assuming a two-sided test with an alpha level of 0.05, a power of 80%, and a moderate effect size (Cohen’s d = 0.5). It was determined that approximately 120 neonates per group would be necessary to adequately power the study to detect the hypothesized effects. This sample size was adjusted upward to 130 per group to account for any anticipated missing data or removal of patients for data matching at approximately 8%.

The data analysis performed a comparison of neurological outcomes between the intervention and control groups. Statistical methods were employed to assess the efficacy of EPO treatment in improving these outcomes. Data management and analysis were conducted utilizing the statistical software SPSS version 26.0 (SPSS Inc., Chicago, IL, USA). The Kolmogorov–Smirnov test was used to determine the normality of data. Normally distributed continuous variables were represented as the mean ± standard deviation (SD), while categorical variables were expressed in terms of frequencies and percentages. Student’s *t*-test was used for comparing two means between normally distributed data and the Mann–Whitney U-test was used for non-gaussian data, respectively. The chi-square test was utilized for the categorical variables. A Pearson correlation was calculated to test associations between continuous variables, and a Spearman’s correlation coefficient was included for categorical variables. A *p*-value threshold of less than 0.05 was set for statistical significance. A Bonferroni correction was applied in the case of multiple comparisons. All results were double-checked to ensure accuracy and reliability.

## 3. Results

### 3.1. Background Characteristics

The analysis revealed no significant differences in mean gestational age between the two groups, with the EPO group having a GA of 34.4 weeks compared with 34.5 weeks in the No EPO group (*p* = 0.804). The distribution across various prematurity categories (extremely preterm to normal) was also not significantly different (*p* = 0.111). Similarly, birth weight comparisons showed no significant difference between the groups, with the EPO group averaging 2333.0 g and the No EPO group averaging 2251.0 g (*p* = 0.399).

Sex distribution and APGAR scores at 1 and 5 min post-birth also showed no statistically significant differences between the two groups. The EPO group had a composition of 61.2% male neonates compared with 54.6% in the No EPO group (*p* = 0.294). APGAR scores at 1 min (EPO: 6.8, No EPO: 7.0; *p* = 0.316) and at 5 min (EPO: 7.4, No EPO: 7.5; *p* = 0.528) were comparable, as presented in Table 1.

### 3.2. Laboratory Results

One of the key findings was the significant difference in LDH levels between the two groups during the early postnatal days. LDH levels on days 1–3 were higher in the EPO group (median 695.0 U/L) compared with the No EPO group (median 679.0 U/L) with a *p*-value of 0.025. This trend reversed by days 7–10, where the EPO group showed significantly lower LDH levels (median 442.0 U/L) than the No EPO group (median 601.5 U/L), with a *p*-value of less than 0.001.

Further significant differences were observed in blood glucose levels, urea, creatinine, CPK, CRP, and PCT, all showing lower median values in the EPO group compared with the No EPO group, with *p*-values of less than 0.001. Notably, erythropoietin levels at 1 week and 3 weeks were significantly higher in the EPO group (27.7 mUI/mL vs. 23.5 mUI/mL in the no EPO group), aligning with the administration of EPO therapy. Meanwhile, hematocrit and hemoglobin levels did not differ significantly; the EPO group exhibited a higher median APTT value (54.0 s) compared with the No EPO group (38.0 s), with a *p*-value of less than 0.001, suggesting a potential influence of EPO therapy on the coagulation pathway (Table 2).

### 3.3. Clinical Outcomes at 1 Week

The incidence of ventriculomegaly was found to be similar in both groups, with an occurrence of 0.8% (1 out of 121) in the EPO group and 0.8% (1 out of 130) in the No EPO group, resulting in a non-significant *p*-value of 0.959. The EPO group had 13 (10.7%) cases of mild HIE, 35 (28.9%) cases of moderate HIE, and 18 (14.9%) cases of severe HIE. In comparison, the No EPO group had 23 (17.7%) mild, 34 (26.2%) moderate, and 34 (26.2%) severe cases (*p* = 0.147).

Intraventricular hemorrhage showed no significant difference between the two groups, with Grade 1 IVH at 14.0% in the EPO group and 13.1% in the No EPO group. The proportions of Grade 2, Grade 3, and Grade 4 IVH were also comparable, with *p*-values indicating no significant differences (*p* = 0.142). Additionally, the rates of apnea were similar in both groups (EPO: 39.7%, No EPO: 41.5%, *p* = 0.100). However, a statistically significant difference was observed in the incidence of bradycardia, being higher in the EPO group (41.3%) compared with the No EPO group (26.9%) with a *p*-value of 0.016, as presented in Table 3.

A significant finding was observed in the incidence of ventriculomegaly at four weeks. In the EPO group, there were no cases (0.0%) of ventriculomegaly, while the No EPO group exhibited a 3.8% incidence (5 out of 130 neonates), resulting in a statistically significant difference with a *p*-value of 0.029. Regarding HIE at week four, the EPO group had lower incidences of moderate (5.0% vs. 20.8%) and severe (1.7% vs. 16.2%) HIE compared with the No EPO group, with a highly significant *p*-value of 0.001.

IVH rates at four weeks also differed significantly between the two groups, while the rates of Grade 1 IVH were similar. The EPO group had significantly lower rates of Grade 2, 3, and 4 IVH compared with the No EPO group (*p* = 0.046), suggesting a potential role of EPO in reducing the risk of severe IVH. The EPO group had a significantly higher rate of status post-intraventricular hemorrhage (23.1% vs. 12.3%, *p* = 0.024) and status post-hypoxic-ischemic encephalopathy (30.6% vs. 11.5%, *p* = 0.002) compared with the No EPO group (Table 4).

### 3.4. Clinical Outcomes at 4 Weeks

The incidence of HIE showed a significant reduction in both groups. In the EPO group, the prevalence decreased from 54.5% (66 out of 121) in the first week to 22.3% (27 out of 121) in the fourth week. Similarly, in the No EPO group, HIE cases reduced from 70.0% (91 out of 130) in the first week to 37.7% (49 out of 130) in the fourth week (*p*-value < 0.001), indicating a substantial decrease in HIE cases over time regardless of EPO therapy.

Furthermore, the study assessed the incidence of IVH over the four-week period. In the EPO group, there was a decrease in IVH cases from 53.7% (65 out of 121) in the first week to 21.5% (26 out of 121) in the fourth week. The No EPO group also showed a reduction from 58.5% (76 out of 130) to 39.2% (51 out of 130). These reductions were statistically significant in both groups (*p* < 0.001), suggesting an overall improvement in IVH cases over time, as presented in Table 5.

### 3.5. Statistical Analyses

One of the key findings was the significant negative correlation between gestational age (GA) and the incidence of HIE and IIVH. GA showed a strong negative correlation with HIE+ (r = −0.376) and IVH+ (r = −0.341), indicating that lower gestational ages were associated with higher incidences of both HIE and IVH. Additionally, hemoglobin (Hb) levels were significantly negatively correlated with HIE+ (r = −0.398) and IVH+ (r = −0.379), suggesting that lower hemoglobin levels in neonates might be associated with an increased risk of these neurodevelopmental complications.

**Figure 1:**
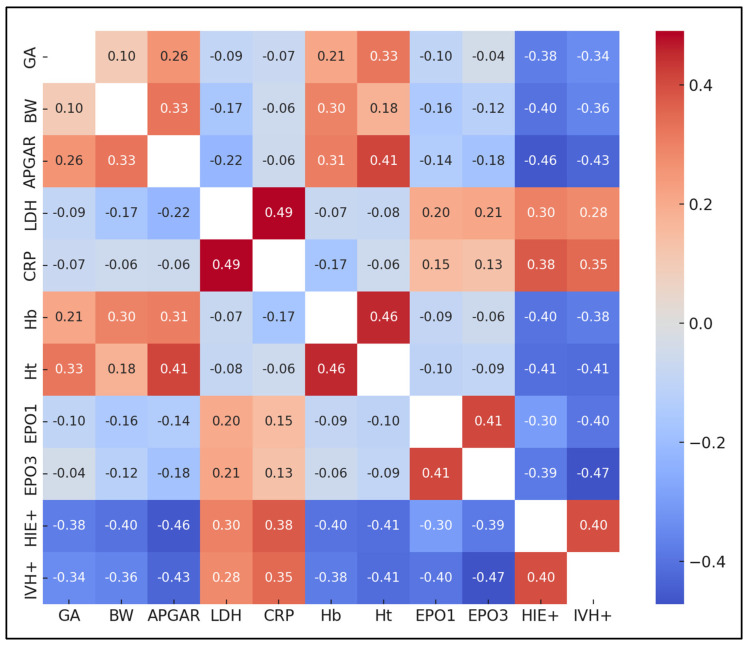
Correlation matrix.

Another notable finding was the significant positive correlation between LDH levels and both HIE+ (r = 0.301) and CRP levels (r = 0.491). This suggests that higher LDH levels were associated with an increased incidence of HIE and higher levels of CRP, an inflammation marker. Furthermore, a strong negative correlation was observed between APGAR scores and HIE+ (r = −0.460) and IVH+ (r = −0.429), indicating that lower APGAR scores at birth were significantly associated with higher risks of HIE and IVH in neonates, as presented in Figure 1.

A significant finding from the analysis was the impact of early EPO administration (within 48 h of birth) on the severity of HIE. The beta coefficient for early EPO administration was −0.38, indicating a significant decrease in HIE severity with early EPO therapy (95% CI: −0.57 to −0.19, *p* = 0.001). This result suggested that early EPO treatment was associated with a marked reduction in HIE severity, reinforcing the potential neuroprotective role of EPO in neonatal care.

Gestational age (GA) and birth weight (BW) were also significant predictors of HIE severity. Extremely preterm GA and very preterm GA were associated with increased severity of HIE, with beta coefficients of 0.72 (*p* = 0.005) and 0.47 (*p* = 0.011), respectively. Similarly, extremely low BW and very low BW were significant predictors of increased HIE severity, with beta coefficients of 0.55 (*p* = 0.002) and 0.31 (*p* = 0.007), respectively, suggesting that lower birth weights were associated with a higher severity of HIE. Furthermore, low hemoglobin levels were significantly associated with increased HIE severity, with a beta coefficient of 0.27 (*p* = 0.028), as seen in Table 6.

## 4. Discussion

The current study revealed significant findings that critically inform our understanding of neonatal care, particularly in the context of neurodevelopmental challenges. An important finding was specifically EPO therapy initiated within the first 48 h being associated with a lower severity of HIE. Moreover, the study presented noteworthy correlations, notably between lower gestational ages and increased incidences of HIE and IVH and between lower hemoglobin levels and the severity of these conditions.

The implications of these findings are profound, especially in neonatal intensive care settings. The statistically significant reduction of IVH and HIE severity with early EPO administration within the first 48 h post-birth suggests a potential management option for at-risk neonates: using a dosage of EPO beta of 1000 U/kg/dose administered subcutaneously, with an escalated dose of 2000 U/kg if required. Furthermore, the study’s insights into the correlations between various neonatal health parameters such as gestational age, birth weight, hemoglobin levels, and the risks of HIE and IVH underscore the complexity of neonatal care.

Similarly to our findings, a recent study discovered that administering multiple high doses of erythropoietin alongside therapeutic hypothermia to term and near-term newborn infants with moderate or severe hypoxic-ischemic encephalopathy did not significantly alter the rate of death or neurodevelopmental impairment when evaluated at the age of two to three years [31,32]. This finding was unexpected, as smaller trials had previously suggested that erythropoietin was both a safe and effective treatment method. The studies also observed that infants who were treated with erythropoietin had a higher likelihood of experiencing at least one serious adverse event and incurred a greater number of serious adverse events in comparison with those who received a placebo [33]. These results stand in contrast to earlier research, which had researched into the safety and efficacy of erythropoietin using complications such as retinopathy of prematurity, suggesting that there were no higher risks of ROP after EPO compared with placebo groups [34].

Nevertheless, EPO use in neonatal care can also be associated with other management options meant to prevent neurological complications, such as induced hypothermia. However, one study on the usage of erythropoietin in the absence of hypothermia has been associated with improved histological and functional outcomes across various animal models of neonatal hypoxic-ischemic brain injury [35]. Nevertheless, the efficacy of erythropoietin in combination with hypothermia has yielded inconsistent results in preclinical studies. While some benefits were observed in nonhuman primates, these results were not replicated in other animals such as rodents, piglets, or sheep [36,37]. The concurrent use of hypothermia in these trials may have diminished the potential additional benefits of erythropoietin, as both treatments are thought to activate similar neuroprotective pathways during the acute phase of hypoxic-ischemic injury, including the reduction of apoptotic, inflammatory, and excitotoxic damage [38].

The large, randomized, placebo-controlled trial cast doubt on the routine clinical practice of administering high doses of erythropoietin to infants undergoing therapeutic hypothermia for HIE, a practice reported in more than 25% of hospitals in certain countries [39,40]. Possible reasons for the absence of positive outcomes could include the adverse effects of erythropoietin given early in the injury process when paired with therapeutic hypothermia; and the possibility of suboptimal dosing or timing of administration, as it is suggested that later doses could be more efficacious in contrast to our approach of early EPO administration before 48 h post-birth [41]. Moreover, there is variance in the injury mechanisms between the preclinical models and actual human cases of hypoxic-ischemic encephalopathy. Nevertheless, in our study, hypothermia was not used as a combined treatment with EPO.

The recent findings regarding the safety of administering multiple high doses of erythropoietin in neonates offer a stark contrast to earlier studies that had endorsed the treatment’s safety [42]. In another major study by Wu et al. [43], no serious adverse event was significantly more frequent in the erythropoietin group compared with the placebo group associated with long-term erythropoietin use in adults, such as hypertension, thrombosis, and polycythemia. A parallel could be drawn with a study in adults suffering from ischemic stroke, where those treated with high doses of erythropoietin experienced a higher mortality rate; however, the study could not pinpoint a singular cause for this outcome [44].

Although our study had a sufficient sample size, it did not follow the patients for long-term neurological and behavioral outcomes due to the retrospective nature of the study. In parallel, another unexpected observation from a recent study was an increased incidence of behavioral abnormalities in two-year-old children who had been treated with erythropoietin compared with those who received a placebo [43]. However, it is important to note that these findings were not adjusted for the multiplicity of testing. The study also did not find significant differences between the two groups in terms of brain MRI findings or functional outcomes, which underscores the complexity of determining the efficacy and safety of erythropoietin treatment in neonates.

In the field of preclinical models, erythropoietin has been shown to promote the regeneration of brain tissue after the acute phase of hypoxic-ischemic injury. It accomplishes this by supporting processes such as neurogenesis, oligodendrocyte genesis, and angiogenesis as well as by increasing the production of growth factors like brain-derived neurotrophic factor [6,45,46]. These later cytoprotective effects might clarify why erythropoietin has been observed to be neuroprotective in rodent models, even when treatment is initiated days after a focal ischemic stroke [43]. Current research efforts are focused on determining whether delayed administration of erythropoietin could offer benefits to term infants who have experienced arterial ischemic stroke [47] and to premature infants who have suffered from intraventricular brain hemorrhage [48] in comparison with our hypothesis that early EPO would benefit infants with IVH and HIE. However, our study’s findings on the early administration of EPO in infants with HIE and IVH underscore the therapeutic potential of early EPO in promoting critical processes like neurogenesis and angiogenesis, mechanisms that are well supported by preclinical models.

The administration of EPO in neonates, particularly those with very low birth weight or experiencing hypoxic conditions, has been researched for its potential neuroprotective effects. Studies have indicated that EPO could support brain development and protect against brain injury in such populations, which could indirectly influence developmental outcomes like head circumference. For instance, erythropoietin’s neuroprotective properties have been explored in various preclinical and clinical settings, highlighting its capacity to support neuronal survival and reduce the impacts of brain injuries caused by conditions like hypoxia [42]. Moreover, the growth and development of head circumference in preterm and low birth weight infants, used as an indicator of overall brain and cognitive development, could be positively affected by interventions like EPO. While direct studies focusing specifically on EPO’s impact on head circumference are limited, the broader implications of its neuroprotective effects suggest a potential benefit that warrants further exploration in detailed clinical trials [49]. Although the current study followed a rigorous methodology, there are multifaceted limitations that arise, with the most significant being its retrospective observational design that captured data from our neonatal cohort but could not conclusively determine the causality between EPO timing and dose administration and neurodevelopmental outcomes. The fixed dosing regimen of erythropoietin within the first 48 h post-birth, although based on the premise of early intervention, did not allow for adjustment according to individual patient responses, potentially overlooking optimal therapeutic windows. A potential confounding factor in our study is the variability in pre-existing health conditions and management of neonates prior to and during the study. Differences in the severity and treatment of conditions like HIE and IVH between the EPO-treated and control groups could influence outcomes independently of EPO’s effects. Furthermore, the study’s assessments at only two time points may not have fully captured the evolving nature of neonatal neurological development due to its retrospective design, which can limit the ability to determine causality between early EPO administration and the risk of IVH and HIE or the effect on these neurological complications, necessitating a more longitudinal approach to discern the long-term efficacy and safety of erythropoietin therapy in this vulnerable population.

## 5. Conclusions

In conclusion, the current study supports the evidence that early EPO administration within the first 48 h of birth plays a significant role in lowering the severity of neonatal complications such as IVH and HIE. The substantial reduction in the severity of these conditions, indicated by significant statistical measures, underlines the therapeutic potential of EPO in neonatal care. Moreover, the study brings important new contributions to the field by highlighting key correlations that deepen our understanding of neonatal health, particularly the increased vulnerability of extremely preterm infants and those with lower birth weights to severe HIE and IVH. These insights are invaluable for advancing neonatal clinical practices and research, emphasizing the need for early intervention and comprehensive monitoring in managing at-risk neonates. Nevertheless, future prospective studies should confirm and validate our findings.

## Figures and Tables

**Table 1:** Background characteristics of neonates.

Variables	EPO (*n* = 121)	No EPO (*n* = 130)	*p*-Value *
GA (mean ± SD)	34.4 ± 3.3	34.5 ± 3.1	0.804
GA			0.111
Extremely preterm	6 (5.0%)	5 (3.8%)	
Very preterm	24 (19.8%)	11 (8.5%)	
Moderate preterm	23 (19.0%)	26 (20.0%)	
Late preterm	54 (44.6%)	72 (55.4%)	
Normal	14 (11.6%)	16 (12.3%)	
BW, grams (mean ± SD)	2333.0 ± 840.3	2251.0 ± 696.0	0.399
BW			0.978
Extremely low	2 (1.7%)	3 (2.3%)	
Very low	14 (11.6%)	15 (11.5%)	
Low	61 (50.4%)	67 (51.5%)	
Normal	44 (36.4%)	45 (34.6%)	
Sex (n, %)			0.294
Male	74 (61.2%)	71 (54.6%)	
Female	47 (38.8%)	59 (45.4%)	
APGAR score 1min (mean ± SD)	6.8 ± 1.9	7.0 ± 1.2	0.316
APGAR score 1min <8 (n, %)	69 (57.0%)	88 (67.7%)	0.081
APGAR score 5min (mean ± SD)	7.4 ± 1.4	7.5 ± 1.1	0.528
APGAR score 5min <8 (n, %)	62 (51.2%)	62 (47.7%)	0.574

* Chi-square or Fisher’s exact test; SD—standard deviation; BW—birth weight, GA—gestational age; APGAR—appearance, pulse, grimace, activity, respiration; EPO—erythropoietin.

**Table 2:** Laboratory analysis.

Variables, Median (IQR)	EPO (*n* = 121)	No EPO (*n* = 130)	*p*-Value *
LDH, days 1–3 (255–600 U/L)	695.0 (513.5–933.0)	679.0 (435.8–920.8)	0.025
LDH, days 7–10 (255–600 U/L)	442.0 (317.0–624.0)	601.5 (399.5–890.0)	<0.001
Blood glucose levels (3.88–6.38 mmol/L)	3.4 (2.5–4.1)	3.8 (2.9–4.7)	<0.001
Urea (1.4–8.3)	4.3 (3.0–5.8)	5.2 (3.6–7.1)	<0.001
Creatinine (21–75 µmol/L)	63.0 (45.5–79.5)	76.5 (59.5–87.3)	<0.001
CPK (24–228 U/L)	233.0 (156.5–347.5)	187.5 (109.0–281.5)	<0.001
CRP, day 1 (0–5 mg/L)	3.1 (1.1–8.8)	6.8 (2.8–15.8)	<0.001
CRP, day 3 (0–5 mg/L)	2.5 (1.0–9.5)	4.8 (2.2–11.5)	<0.001
PCT, day 1 (0–0.5 ng/mL)	2.5 (0.6–13.4)	2.8 (0.5–8.8)	0.262
PCT, day 3 (0–0.5 ng/mL)	0.5 (0.2–3.6)	2.3 (0.5–5.2)	<0.001
Blood pH (7.35–7.45)	7.4 (7.3–7.8)	7.3 (7.3–7.4)	0.307
PCO2 (35–46 mmHg)	39.0 (37.0–45.0)	42.0 (38.0–46.0)	<0.001
PO2 (70–100 mmHg)	65.0 (58.0–70.0)	66.5 (58.0–74.0)	<0.001
Lactic acid (0.50–2.20 mmol/L)	3.2 (2.2–5.0)	3.2 (2.1–5.1)	0.554
EPO, 1 week (4.3–29.0 mUI/mL)	7.2 (4.2–13.1)	5.1 (3.0–12.1)	0.027
EPO, 3 weeks (4.3–29.0 mUI/mL)	27.7 (19.5–38.4)	23.5 (16.4–35.2)	0.006
Hematocrit, day 1 (53–65%)	43.8 (37.8–49.0)	43.0 (37.0–47.0)	0.052
Hemoglobin, day 1 (15.5–21.5 g/dL)	15.2 (13.4–16.9)	15.0 (13.8–16.7)	0.647
RBCs (4.7–6.3 × 10^6^/uL)	4.2 (3.8–4.7)	4.0 (3.8–4.4)	0.090
APTT (25–50 s)	54.0 (34.0–67.0)	38.0 (33.8–43.0)	<0.001
PT (9.5–14 s)	13.9 (12.6–15.9)	13.0 (12.0–15.0)	0.063

* Mann–Whitney U-test; SD—standard deviation; LDH—lactate dehydrogenase; CPK—creatine phosphokinase; CRP—C-reactive protein; PCT—procalcitonin; EPO—erythropoietin; RBCs—red blood cells; APTT—activated partial thromboplastin time; PT—prothrombin; IQR—interquartile range.

**Table 3:** Outcomes and complications at 1 week.

Variables (n, %)	EPO (*n* = 121)	No EPO (*n* = 130)	*p*-Value *
Ventriculomegaly—Week 1	1 (0.8%)	1 (0.8%)	0.959
HIE—Week 1			0.147
Mild	13 (10.7%)	23 (17.7%)	
Moderate	35 (28.9%)	34 (26.2%)	
Severe	18 (14.9%)	34 (26.2%)	
IVH—Week 1			0.142
Grade 1	17 (14.0%)	17 (13.1%)	
Grade 2	44 (36.4%)	45 (34.6%)	
Grade 3	3 (2.5%)	13 (10.0%)	
Grade 4	1 (0.8%)	1 (0.8%)	
Apnea	48 (39.7%)	54 (41.5%)	0.100
Bradycardia	50 (41.3%)	35 (26.9%)	0.016

* Chi-square test; EPO—erythropoietin; IVH—intraventricular hemorrhage; HIE—hypoxic-ischemic encephalopathy.

**Table 4:** Outcomes and complications at 4 weeks.

Variables (n, %)	EPO (*n* = 121)	No EPO (*n* = 130)	*p*-Value *
Ventriculomegaly—Week 4	0 (0.0%)	5 (3.8%)	0.029
HIE—Week 4			0.001
Mild	19 (15.7%)	21 (16.2%)	
Moderate	6 (5.0%)	27 (20.8%)	
Severe	2 (1.7%)	21 (16.2%)	
IVH—Week 4			0.046
Grade 1	20 (16.5%)	23 (17.7%)	
Grade 2	5 (4.1%)	17 (13.1%)	
Grade 3	1 (0.8%)	7 (5.4%)	
Grade 4	0 (0.0%)	4 (3.1%)	
Hydrocephalus—Week 4	2 (1.7%)	1 (0.8%)	0.519
Status post-intraventricular hemorrhage—Week 4	28 (23.1%)	16 (12.3%)	0.024
Status post-hypoxic-ischemic encephalopathy—Week 4	37 (30.6%)	15 (11.5%)	0.002

* Chi-square test; EPO—erythropoietin; IVH—intraventricular hemorrhage; HIE—hypoxic-ischemic encephalopathy.

**Table 5:** Outcomes and complications at 4 weeks.

Variables (n, %)	EPO Group Week 1	EPO Group Week 4	No EPO Group Week 1	No EPO Group Week 4	Change in EPO Group (*p*-Value) *	Change in No EPO Group (*p*-Value) *
Ventriculomegaly	1 (0.8%)	0 (0.0%)	1 (0.8%)	5 (3.8%)	0.962	0.209
HIE (Mild/Moderate/Severe)	66 (54.5%)	27 (22.3%)	91 (70.0%)	49 (37.7%)	<0.001	<0.001
IVH (Grade 1–4)	65 (53.7%)	26 (21.5%)	76 (58.5%)	51 (39.2%)	<0.001	<0.001

* Chi-square test; EPO—erythropoietin; IVH—intraventricular hemorrhage; HIE—hypoxic-ischemic encephalopathy.

**Table 6:** Multiple linear regression analysis for the severity of HIE.

Predictor Variable	Beta Coefficient	95% CI	*p*-Value
Early EPO administration (<48h)	−0.38 *	−0.57–−0.19	0.001 *
No EPO (reference)	–	–	–
Extremely preterm GA	0.72 *	0.31–1.13	0.005 *
Very preterm GA	0.47 *	0.18–0.76	0.011 *
Moderate–Late preterm GA	0.29	−0.02–0.60	0.065
Normal GA (reference)	–	–	–
Extremely low BW	0.55 *	0.23–0.87	0.002 *
Very low BW	0.31 *	0.09–0.53	0.007 *
Low BW	0.2	−0.04–0.44	0.166
Normal BW (reference)	–	–	–
Low hemoglobin level	0.27 *	0.05–0.49	0.028 *
Normal hemoglobin level (reference)	–	–	–

Adjusted R^2^ = 0.410; Asterisks (*) indicate statistically significant results; CI—confidence interval; EPO—erythropoietin; IVH—intraventricular hemorrhage; HIE—hypoxic-ischemic encephalopathy; OR—odds ratio.

## Data Availability

Informed consent was obtained from the parents and legal guardians of all neonates involved in the study.
